# The Economic Impact of Hurricane Evacuations on a Coastal Georgia Hospital: A Case Study

**DOI:** 10.3389/fpubh.2019.00149

**Published:** 2019-06-11

**Authors:** Samir P. Desai, Jimmy Gordon, Curtis Andrew Harris

**Affiliations:** ^1^College of Public Health, Institute of Disaster Management, University of Georgia, Athens, GA, United States; ^2^Memorial Health University Medical Center, Savannah, GA, United States

**Keywords:** hospital evacuation, coastal hospital, costs, vulnerable populations, economic impact of hurricanes hurricane evacuation

## Abstract

Coastal hospitals are often faced with the challenging decision to either evacuate or shelter-in-place in anticipation of a hurricane predicted to make landfall. The costs associated with hospital evacuation not only include transportation of patients to inland areas, but also the loss of revenue due to interruption of regular operations and the cost of potential damage to the hospital's infrastructure. Financial data provided by Memorial Health University Medical Center (MUMC, Savannah, Georgia) such as average inpatient and outpatient revenues, personnel wages, and transportation costs, were used to estimate the potential economic impact of hurricane evacuations on a coastal hospital. The results indicate that even prior to the arrival of tropical storm force winds, the hospital will incur an estimated total expenditure of approximately $9.5 million which includes evacuation expenses and loss of revenue due to disruption of regular services. In case the hurricane makes landfall, revenue losses will continue to accumulate until the hospital is able to resume regular operations. The cost of relocating patients back to MUMC after the hurricane event and the cost of any hurricane-related damage to the hospital infrastructure must also be taken into consideration. In conclusion, even though hospital evacuation for hurricanes may be unavoidable in certain circumstances, the financial burden placed upon the hospital may be mitigated to a certain extent by forward planning, infrastructure upgrades, and the rapid resumption of regular hospital function.

## Introduction

The economic damage inflicted by hurricanes has increased dramatically with over two-thirds of the costliest hurricanes occurring in the past 25 years (1). Hurricane Katrina was one of the most devastating natural disasters to strike the United States as well as one of the costliest hurricanes on record with a total damage estimate of $108 billion (2). Additionally, victims of Hurricanes Katrina and Rita combined received more than $110 billion in rescue, recovery, and reconstruction operations by the US government (3). Scientific advancements in weather sensing and reporting technology over the past few decades have led to an increased ability to track hurricanes with a consequent rise in hurricane warnings and evacuation orders along the coastal areas. During the 2000 to 2006-time period, an average of 3.5 hurricane storm warnings were declared in the United States per year, affecting approximately 417 miles of coastline per storm. The potential annual costs of evacuating 417 miles of coastline with each hurricane warning would be approximately $1.5 billion. Typically, hurricane force winds impact only about 25% of the area under warning, conceivably resulting in over $1 billion in unnecessary expenditures (4).

While specific evacuation plans for coastal areas depend upon their respective geographic locations and transportation characteristics, most states follow a two-tiered approach. Evacuation planning and response are carried out at the local (e.g., county or city) level and state-level agencies serve to coordinate these activities (5). Georgia is a “home rule” state wherein the cities, municipalities and/or counties have the ability to pass governance laws, including evacuation timelines, at their discretion. Although the minimum advance notification time for evacuation of coastal populations is 24–36 h prior to the arrival of tropical force winds, evacuation activities for healthcare institutions may begin earlier considering the vulnerable state of its population and/or the location of the healthcare facility which may lie below sea level with limited egress. Hospital evacuation in the event of a hurricane is an inherently complex task due to the vulnerability of its population and the associated costs, both monetary as well as in terms of human lives at stake. Typically, hospital administrators have to make the choice between either evacuating or sheltering in place. In each situation, the logistics of managing a highly diverse patient population in terms of their acuity levels and treatment modalities can present a daunting challenge. For coastal hospitals in particular, the decision to evacuate can cause great financial hardship, in terms of transportation costs loss of revenue.

The inherent uncertainty associated with storm forecasts further complicates the decision-making process for hospitals (4). At 72 h prior to the forecasted hurricane making landfall, the strike probability is ~10–15% resulting in a high likelihood of an unnecessary evacuation. Patient evacuation necessitates securing appropriate transportation (either emergent or non-emergent), procuring available bed space (requires a physician to physician and nurse to nurse handoff), and obtaining sufficient resources (e.g., staff, medical supplies) to ensure a smooth transition from one healthcare facility to another. The decision to shelter in place requires the availability of medical and non-medical staff and supplies, food, water, and a robust communications system to ride out the storm until help arrives from the outside, all of which has associated costs that must be accounted for during preparedness and mitigation phases.

In this manuscript, the authors offer an overview of the financial costs (including revenue loss) that may be encountered by Memorial Health University Medical Center (MUMC) in coastal Georgia due to a hurricane evacuation.

### Overview of Memorial University Medical Center

Memorial University Medical Center (MUMC) is a non-profit, 604-bed tertiary care hospital located in Savannah, Georgia. It began operating in 1955 and has since become a regional referral center for cardiac care, cancer care, trauma, pediatrics, high-risk obstetrics, and neonatology. The hospital includes the region's only Level 1 trauma center and the region's only pediatric hospital. MUMC is a two-state healthcare organization serving a 35-county area in southeast Georgia and southern South Carolina, and is one of the largest employers in the region, with nearly 5,000 employees (6).

## Methods

MUMC has a robust emergency preparedness plan for hurricane evacuations. It includes a detailed checklist of evacuation activities that begin as far out as 120 h (5 days) before the arrival of tropical storm force winds. MUMC staff are divided into 3 separate teams (Teams A, B, and C), each of which has a well-defined role in the evacuation process. Teams A and B are composed of registered nurses (RNs), licensed practical nurses (LPNs), primary care providers (PCPs), and support staff. Team C is composed of only of RNs and LPNs.

Team A is charged with sheltering-in-place at MUMC during the storm and caring for high acuity patients that are too fragile to transport. Team A members report to the hospital 48 h prior to tropical storm force winds. Team B is allowed to disperse 48–36 h before the arrival of storm force winds with the expectation that they will return within 12 h during the recovery phase. Team C evacuates along with the patients and accompanies them to the receiving facility. All teams work in 12 h shifts.

Events that either directly pertain to patient evacuation, or are relevant to cost calculations, begin with the early discharge of inpatients that are ambulatory and in stable medical condition. This occurs at 120 h (−5 days) prior to the arrival of tropical storm force winds. At 96 h (−4 days), individuals with special needs such as neonates, behavioral and pediatric patients are evacuated via appropriate means of transportation. The regular evacuation of patients begins at the 72 h (−3 days) mark. As mentioned above, these patients are accompanied by members of Team C to the receiving facility/facilities located further inland.

### Cost Calculations

Financial data were collected from MUMC for a potential hurricane evacuation under two main categories—loss of revenue and cost of evacuation as shown in Table 1a.

**Table 1a:** Cost collection matrix.

**Activity**	**Methodology**
Early discharge of ambulatory inpatients	Average daily inpatient revenue **×** Average inpatient duration of stay (days) **×** Average number of patients discharged
**REVENUE LOSS DUE TO CANCELLATION OF MAJOR ACTIVITIES**	
Inpatient admissions	Average per patient revenue **×** Average number of patients/visits per day
Outpatient procedures	
Elective surgeries	
ER services	
**EVACUATION OF SPECIAL NEEDS PATIENTS PRIOR TO REGULAR EVACUATION**	
Neonates	Number of patients **×** Transportation cost **+** Hourly wage for 2 EMTs
Pediatrics	
Behavioral	
Regular evacuation	Number of patients **×** Transportation cost **+** Hourly wage for 2 EMTs
Lodging + Food and Wages for Team C (per day)	Number of personnel **×** Hourly wage **×** 12 h **+** Food and Lodging cost
Wages for Team A (per day)	Number of personnel **×** Hourly wage **×** 12 h

Loss of revenue includes major hospital activities that generate substantial earnings such as inpatient admissions, outpatient procedures, elective surgeries, and emergency room (ER) services. Cancellation/postponement of major hospital activities begins at 120 h prior with complete cessation of services at 96–72 h before the storm. For the sake of brevity, it has been assumed that MUMC will suffer from loss of revenue for all major activities starting at the 120 h time point. The average number of daily patient admissions/visits along with the average revenue generated for each category is listed in Table 1b.

**Table 1b:** Average daily patient visits and revenue.

**Service type**	**Average per patient revenue**	**Average # of patients per day**
In-patient	$2,184	72
Out patient	$741	646
ER visit	$263	272
Elective surgery	$6,828	41

The cost of evacuation includes expenditures incurred for transferring patients via Emergency Medical Services (EMS) (e.g., Advanced Life Support (ALS) or Basic Life Support (BLS) ambulances), or by non-emergent transport (e.g., wheelchair van or bus). Medicare reimbursement rates in the Metro Atlanta service area for ALS ($429/trip) and BLS ($362/trip) ambulances were used to calculate transportation costs (7). Transportation by bus was valued at a flat rate of $2,800 as per MUMC.

Mean hourly wage information for personnel on Teams A and C, and for the Emergency Medical Technicians (EMTs) staffing the ALS and BLS ambulances were obtained from the Bureau of Labor Statistics (8). Evacuation costs also take into account the cost of lodging and food for Team C members which were obtained from the United States General Services Administration (9).

## Results

The final expenditures associated with hurricane evacuation at MUMC can be divided into two groups (Table 2). One-time costs include revenue loss due to early discharge of inpatients and the actual cost of evacuation (transportation). Recurring costs include revenue loss due to interruption of major hospital activities, wages for Teams A and C, and food/lodging costs for Team C. These are calculated on a per day basis.

**Table 2:** Cost calculations (one-time and recurring).

**Activity**	**Methodology**	**Calculation**	**Amount**
**ONE-TIME COSTS**			
Early discharge of ambulatory inpatients	Average daily inpatient revenue **×** Average inpatient duration of stay (days) **×** Average number of patients discharged	$2,184 × 6 days × 306 patients	$4,009,824
**Evacuation of special needs patients prior to regular evacuation**			
Neonates	Number of patients **×** Transportation cost + EMT wages	(56 patients × $429) + (2 EMTs × $16.88 × 140 h)	$28,750.40
Pediatrics		(15 patients × $429) + (2 EMTs × $16.88 × 37.5 h)	$7,701.00
Behavioral	Transportation cost (by bus)	15 patients @ $2,800	$2,800
**Regular evacuation**			
Regular transportation	Transportation cost (by bus)	15 patients @ $2,800	$2,800
BLS	Number of patients **×** Transportation cost + EMT wages	(50 patients × $362) + (2 EMTs × $16.88 × 125 h)	$22,295.00
ALS		(50 patients × $429) + (2 EMTs × $16.88 × 125 h)	$25,684.50
Total			$4,099,854.9
**RECURRING COSTS**			
**Revenue loss due to cancellation of major activities (per day)**			
Inpatient admissions	Average per patient revenue **×** Average number of patients/visits per day	$2,184 × 72 patients/day	$157,248
Outpatient procedures		$741 × 646 procedures/day	$478,686
Elective surgeries		$6,828 × 41 surgeries/day	$279,948
ER visits		$263 × 272 visits/day	$71,536
**lodging + food and wages for team C (per day)**			
RN	Number of personnel **×** Hourly wage **×** 12 h + Food and Lodging cost	126 × $33.55 × 12 h + $12,033.00	$62,760.60
LPN		2 × $20.87 × 12 h + $191.00	$691.88
**Wages for team A (per day)**			
RN	Number of personnel **×** Hourly wage **×** 12 h	79 × $33.55 × 12 h	$31,805.40
LPN		3 × $20.87 × 12 h	$751.32
PCP		19 × $91.23 × 12 h	$20,800.44
Support staff		78 × $17.04 × 12 h	$15,949.44
Total			$1,120,177.08

**Figure 1:**
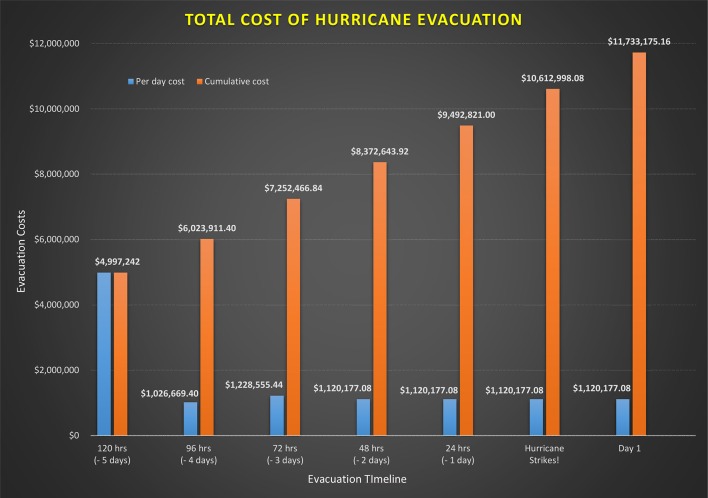
Cumulative costs of hurricane evacuation by timeline.

The total calculated one-time cost of evacuating patients at MUMC is $4,099,854.90. This includes the early discharge of ambulatory patients at 120 h (−5 days), evacuation of special needs patients at 96 h (−4 days), and regular evacuation at 72 h (−3 days). According to MUMC, an average of 306 patients will be discharged early followed by the evacuation of 86 special needs patients, and finally, the evacuation of the remaining 115 patients. The application of recurring costs follows a staggered approach depending upon the evacuation timeline (see Figure 1). For example, revenue loss due to cancellation of major activities begins at 120 h (−5 days). On the other hand, cost of food/lodging and wages for members of Team C will begin at 72 h (−4 days). Similarly, wages for members of Team A will begin to accrue from 48 h (−2 days). Once a recurring cost is introduced, it will continue to accrue for each day thereafter until the all clear signal is provided. The daily cost to MUMC plateaus at $1,120,177.08 at 48 h (−2 days) and remains constant for each following day.

Figure 1 also shows the cumulative cost borne by MUMC at each time point in the evacuation process. Rounding out the numbers to the nearest hundred-thousand, the total cumulative cost increases from $5 million at 120 h (−5 days), $6 million at 96 h (−4 days), $7.3 million at 72 h (−3 days), $8.4 million at 48 h (−2 days), and $9.5 million at 24 h (−1 day).

## Discussion

On average, five hurricanes will impact the United States coastline every 3 years, and two of these hurricanes will be classified as major, that is, category 3 or greater on the Saffir-Simpson Hurricane Scale (10). Georgia is one of the few states in the southeastern United States that has been mostly spared from hurricane activity on land which includes hurricane force winds, the eye of the hurricane actually passing through the coastal area, or both. Although Hurricane Floyd came close to the Georgia coastline in 1999 triggering an evacuation, the last hurricane to make landfall in Georgia was Hurricane David (Category 2) in 1979. However, coastal Georgia is often impacted indirectly by tropical storms that make landfall in the Florida Panhandle. These storms may have low wind velocities, but can bring copious rainfall thereby causing serious flooding issues in the affected areas (11).

During the days leading up to Hurricane Katrina, New Orleans hospitals were in the unenviable position of deciding whether to evacuate or to shelter-in-place, given the general unpredictability associated with hurricane forecasts. While none of the New Orleans area hospitals evacuated in advance of the storm, St. Charles Parish Hospital, located 20 miles west of New Orleans, took an alternate approach. Hospital administrators decided to evacuate their sickest patients by ambulance and the rest by wheelchair accessible school buses. While patients transported by ambulance received an escort from the sheriff's department and arrived at the receiving facility 320 miles away, those transported by bus were stuck in traffic and had to ride out the storm at an emergency shelter in Lafayette, some 120 miles away from St. Charles Parish Hospital (12). Post-Katrina, the Joint Commission accreditation standards were updated to include flood-resistant power supply and monthly capability tests. However, retrofitting existing infrastructure was considered cost-prohibitive and the Joint Commission recommended a more resilient infrastructure for future hospital construction (13). Despite the considerable expenditure involved in retrofitting existing facilities, hospitals could ultimately benefit from increased resiliency during a hurricane and rapid resumption of normal operations in its aftermath.

The above-mentioned dilemma is further aggravated due to the paucity of resources such as personnel, transport vehicles and highway space. An investigation into evacuation practices of hospitals during Hurricane Rita, which struck the US Gulf Coast some 3 weeks after Hurricane Katrina, showed that the most significant factors triggering evacuations were the issuance of mandatory evacuation orders, storm dynamics, and loss of communications (14). Interestingly, of the 275 reported hospital evacuations from 1971 to 1999, hurricanes accounted for only 14% (38) of the evacuation incidents (15).

Previous studies have attempted to measure the public costs of hurricane evacuations. For example, an analyses of estimated household evacuation costs for ocean counties in North Carolina ranged from $1 million to $50 million depending on storm intensity and emergency managements policies (16). Another study provided a quantitative method for estimating hurricane damage costs and found that for Lee county, Florida, the potential public costs range from $4.7 million for a category 1 hurricane to $130 million for a category 5 hurricane (17). However, peer-reviewed literature on assessment of hurricane evacuation costs for hospitals is relatively sparse (18). One study reported that the overall cost associated with the evacuation of three New York hospitals during Hurricane Irene was $13 million, which includes $4 million ascribed to labor and supplies, and $9 million as revenue loss due to cancellation of regular services (19). However, expenditures associated with the evacuation of 947 patients from the New York hospitals was not included in the overall cost estimates. Although difficult to project prospectively, the cost of potential damages to healthcare facilities due to wind or water must also be considered. As per the National Association of Community Health Centers, Hurricane Katrina caused more than $43 million in damages to community health centers in New Orleans alone (20). The costs associated with re-entry (i.e., the process of transporting patients back to MUMC after the storm has abated), should be taken into account as well. It should be noted that certain evacuation costs may be reimbursable under the Stafford Act of 1988 following a presidential declaration of emergency. These include overtime wages for full-time personnel involved in the evacuation effort, equipment costs associated with patient evacuation and re-entry (including transportation), and cost of treatment required to stabilize patients for evacuation (21).

MUMC possesses a property insurance policy with an evacuation endorsement provision which covers additional expenses related to extraordinary events such as a fire or hurricanes. Typically, the evacuation provision comes into effect once the civilian authorities declare a mandatory evacuation. Declaration of mandatory evacuation is based on storm forecasts by the National Hurricane Center which is currently capable of predicting strike locations within about 100 miles and 24 h before storm landfall (5). A 24 h lead time for evacuating large civilian populations is hardly sufficient as illustrated by Hurricane Floyd which resulted in one of the largest evacuations in US history and consequently caused one of its biggest traffic jams. Therefore, evacuation of vulnerable hospital patients alongside the mass exodus of civilians can be a major challenge. However, MUMC's insurance policy is scripted so that the activation of the evacuation provision is not dependent upon the issuance of mandatory evacuation orders. This is due to the fact that MUMC has neonates among its patient population that need to be evacuated prior to the regular evacuation. Major expenses covered by the policy include—Transportation costs (e.g., ambulance and charter bus) and, food and lodging costs for Team C members accompanying the evacuees.

Another issue worth mentioning is the evacuation timeline adopted by a particular healthcare facility. MUMC is a fairly large hospital serving a wide geographic area and would require a longer lead time to prepare for and execute a hurricane evacuation plan. Smaller sized healthcare facilities on the other hand could potentially decrease their revenue loss by downsizing the evacuation timeline. For example, while MUMC begins cancellation/postponement of major hospital activities at 120 h prior to the anticipated arrival of storm force winds, a smaller facility may begin doing so at the 72 h mark.

## Conclusion

The total expenditure for hurricane evacuation at MUMC would be approximately $9.5 million prior to the arrival of tropical storm force winds. This cost estimate could increase exponentially if the hurricane actually makes landfall thereby extending the daily revenue loss. Although evacuation insurance carried by MUMC and potential reimbursement from the Federal Emergency Management Agency (FEMA) might mitigate some of the costs, MUMC would still have to contend with the loss of a fairly large portion of its daily revenue. Coastal area hospitals should consider retrofitting their existing infrastructure in order to ensure continuity of operations, rapid normalization of major hospital activities post-event, and also to minimize damage to the facility.

## Author Contributions

SD is the lead author and is credited with writing the manuscript, as well as analyzing the financial data presented within the manuscript. CH is the second author and is credited with reviewing the entire manuscript, including data analyses. JG is credited with providing the raw financial data as well as guidance on data analyses.

### Conflict of Interest Statement

The authors declare that the research was conducted in the absence of any commercial or financial relationships that could be construed as a potential conflict of interest.
